# Quantifying the Public Health Benefits of Reducing Air Pollution: Critically Assessing the Features and Capabilities of WHO’s AirQ+ and U.S. EPA’s Environmental Benefits Mapping and Analysis Program – Community Edition (BenMAP – CE)

**DOI:** 10.3390/atmos11050516

**Published:** 2020-05-16

**Authors:** Jason D. Sacks, Neal Fann, Sophie Gumy, Ingu Kim, Giulia Ruggeri, Pierpaolo Mudu

**Affiliations:** 1 Center for Public Health and Environmental Assessment, Office of Research and Development, U.S. Environmental Protection Agency, Research Triangle Park, NC 27709;; 2 Office of Air Quality Planning and Standards, Office of Air and Radiation, U.S. Environmental Protection Agency, Research Triangle Park, NC 27709;; 3 Department of Public Health, Environmental and Social Determinants of Health, World Health Organization, Geneva, Switzerland;; 4 European Centre for Environment and Health, World Health Organization Regional Office for Europe, Bonn, Germany;; 5 Department of Public Health, Environmental and Social Determinants of Health, World Health Organization, Geneva, Switzerland;; 6 European Centre for Environment and Health, World Health Organization Regional Office for Europe, Bonn, Germany;

**Keywords:** air quality, PM_2.5_, BenMAP – CE, AirQ+

## Abstract

Scientific evidence spanning experimental and epidemiologic studies has shown that air pollution exposures can lead to a range of health effects. Quantitative approaches that allow for the estimation of the adverse health impacts attributed to air pollution enable researchers and policy analysts to convey the public health impact of poor air quality. Multiple tools are currently available to conduct such analyses, which includes software packages designed by the World Health Organization (WHO): AirQ+, and the U.S. Environmental Protection Agency (U.S. EPA): Environmental Benefits Mapping and Analysis Program – Community Edition (BenMAP – CE), to quantify the number and economic value of air pollution-attributable premature deaths and illnesses. WHO’s AirQ+ and U.S. EPA’s BenMAP – CE are among the most popular tools to quantify these effects as reflected by the hundreds of peer-reviewed publications and technical reports over the past two decades that have employed these tools spanning many countries and multiple continents. Within this paper we conduct an analysis using common input parameters to compare AirQ+ and BenMAP – CE and show that the two software packages well align in the calculation of health impacts. Additionally, we detail the research questions best addressed by each tool.

## 1. Introduction

Poor air quality is one of the leading global risk factors that can contribute to premature death and disability adjusted life years (DALYs) [1,2]. It has been well documented that efforts to reduce air pollution can lead to substantial health benefits, such as reducing premature deaths and the exacerbation or development of a number of respiratory- and cardiovascular-related diseases [2,3]. The assessment of the health impacts of air pollution can be beneficial to not only conveying the public health impact of poor air quality, but also when considering the potential implementation of various air quality policies. Therefore, assessments of the potential health impacts that could be achieved through improvements in air quality represent an important data point for public health and environmental specialists.

To estimate the public health impact of changes in air quality, which includes both the number of premature deaths and illnesses and often their associated economic value, numerous tools of varying complexity have been developed [3–6]. Of these tools, the World Health Organization’s AirQ+ and U.S. Environmental Protection Agency’s (U.S. EPA) Environmental Benefits Mapping and Analysis Program – Community Edition (BenMAP – CE) are among the most popular. Both AirQ+ and BenMAP – CE have been used and reported in an extensive number of peer-reviewed publications and technical reports, which reflects their varying functionality in terms of the types of analyses and questions each can address. The range of countries where applications of AirQ+ and BenMAP – CE were performed covers multiple continents, with the most extensive use in Asia, North America, South America and Europe.

BenMAP was initially released in 2003 and represented the primary tool used by the U.S. EPA to estimate the health and economic benefits of attaining current and potentially future National Ambient Air Quality Standards (NAAQS) [8]. While the tool was used extensively by the U.S. EPA, it was also used in multiple research efforts to estimate the potential public health impacts of improving air quality [9,10]. Building off the original version of BenMAP, starting in 2012, the U.S. EPA transformed the tool into an open-source software platform to allow for it to be more broadly accessible to the global air pollution research community, culminating in the release of BenMAP – CE in 2015 [8]. Like the original version, BenMAP – CE is extensively used by the U.S. EPA in various policy-related analyses, but its use by the broader research community has grown exponentially. This can be attributed to not only BenMAP – CE being freely available, but also the various types of analyses that can be conducted using the tool. As detailed within Sacks et al. [8], data inputs, which can range from fine to coarse resolution in spatial scale and can include both monitored and modeled air quality data, allow for conducting analyses of varying complexity.

While BenMAP was originally developed to support U.S. air quality policy analyses, and only within the last 10 years expanded to have a global reach, WHO developed an Excel-based software tool called AirQ starting in 1999, originally targeting only to European countries. The original aims of AirQ were to: (1) convey to the user the most important and best recognized health effects attributed to air pollution; (2) provide an incentive to collect and analyze data on air pollution (use of nationally/locally available air quality data), (3) share the air quality data with the WHO Regional Office for Europe; and (4) provide easily interpretable results to convey the overall health impact of air pollution, such as Attributable Fraction (AF), attributable morbidity and mortality, and Years of Life Lost (YLLs) estimates [11].

Between 1999 and 2001 this tool was programmed to focus on health impacts attributed to short-term exposures (i.e., daily variations in air quality). From 2001 to 2004, AirQ was tested and expanded to also estimate health impacts attributed to long-term air pollution exposures, which ultimately contributed to the tool being widely used by experts internationally. Although AirQ was gaining wide acceptance within the research community, in the following years WHO recognized the tool needed to be updated to incorporate the latest scientific evidence on the health impacts attributed to air pollution and to respond to the new technological requirements of computer systems. As a result, in 2016 AirQ+ was developed building on the success of AirQ, but with the specific purposes of (1) reflecting the current state of the science on the health effects of air pollution; (2) ensuring that researchers and governmental officials worldwide could have access to a tool to inform and ultimately support actions to improve air quality, and (3) to provide a large audience with an educational tool that includes summaries of the information that needs to be gathered and organized to understand the impacts of air pollution on health. To facilitate the goal of achieving broad acceptance and use of the tool, versions of AirQ+ are currently available in English, French, and Russian with German and Spanish language versions under development.

While BenMAP – CE and AirQ+ have been two of the most extensively used tools to estimate the potential health impacts of changes in air quality, with BenMAP – CE having the additional feature of being able to estimate the potential economic benefits, the two tools have not been evaluated using a similar dataset. Within this paper, we conduct an analysis using a common, hypothetical dataset, to demonstrate how basic analyses are conducted using both BenMAP – CE and AirQ+, and highlight the differences and similarities between the two tools. Specifically, this paper highlights the underlying methodology used to estimate health impacts in both tools, the data preloaded within each tool along with the data that can be provided by the user to tailor analyses, and the differences in the processes used by U.S. EPA and WHO to identify the health impacts to estimate.

## 2. Methods

### Approach to Estimating the Number of Premature Deaths and Illnesses

For this analysis, the tools estimating the number of avoided premature deaths and illnesses attributed to improving PM_2.5_ air quality use a simple algebraic equation often referred to as a health impact function (HIF). As detailed in Equation (1), the HIF calculates counts of premature deaths and illnesses using four input parameters: (1) modeled or monitored air pollutant concentrations; (2) population data; (3) baseline rates of death or disease; and (4) a concentration-response parameter (often referred to as a beta coefficient from an epidemiologic study that measures the risk of a health effect due to a one-unit change in an air pollutant concentration) and is commonly defined as [8]:

(1) $$\Delta \text{Y}=\left(1-e^{-\beta *\Delta \text{C}}\right)*\text{Yo}*\text{Pop}$$

Where ΔY = the estimated number of premature deaths or illnesses, β = the risk estimate (or Beta coefficient) from an epidemiologic study, ΔC = the defined change in the concentration of the air pollutant examined; Yo = the baseline rate (i.e., incidence) of deaths or illnesses; and Pop = the population exposed to air pollution. The HIF allows both AirQ+ and BenMAP – CE to estimate the number of avoidable premature deaths and illnesses that could result from improving air quality over a defined geographic location. While both tools use the same underlying information to estimate the number of premature deaths and illnesses, the data preloaded within each tool and the types of data that can be provided by the user varies (Table 1), which highlights the main differences between to two tools.

### 2.1. Input Parameters

Each of the two tools quantifies the number of “avoided” attributable PM_2.5_-related premature deaths using a common set of input parameters based on data from the city of Budapest, Hungary for the years 2004 – 2006 as detailed in Malmqvist et al. [12]. Throughout this paper, the original, validated data from Budapest represents the location Subregion 1, whereas, the randomly modified data from Budapest is used to represent a hypothetical location, Subregion 2. The hypothesis for the generation of the Subregion 2 dataset was to simulate randomly, but with some assumptions, a fictitious metropolitan area surrounding Budapest, with higher levels of air pollution and a younger population. These data included: ambient PM_2.5_ concentrations; a count of the adult population; the baseline rate of all-cause death; and, a concentration-response parameter for PM_2.5_-related all-cause mortality. The input parameters for each Subregion analysis are detailed in Table 2.

#### 2.1.1. Air Quality Data

We employed two versions of the PM_2.5_ air quality data to serve as an analytical baseline. In the first, defined as Subregion 1, we use validated 24-hour average monitored PM_2.5_ concentration data for the city of Budapest, Hungary for the years 2004 – 2006. In the second, defined as Subregion 2, we randomly modified the PM_2.5_ data for Subregion 1 to simulate a location with overall higher PM_2.5_ concentrations, which helps characterize the sensitivity of the estimated PM_2.5_-attributable deaths to differences in the baseline PM_2.5_ concentration changes. Using the validated PM_2.5_ data for Subregion 1 as a starting point, PM_2.5_ concentrations were increased by 4 μg/m^3^ for all but 15 days. In those 15 days PM_2.5_ data was further modified. The 15 days were identified by extracting 3 months (i.e., May 2004, March 2004, and December 2006) from the entire dataset. Within these three months, 13 days were modified in May 2004, 1 day in March 2004, and 1 day in December 2006. Across these 15 days, values were changed using the following approach: for 10 days the addition of a randomly generated number from 6 to 30 to the original PM_2.5_ concentration for each day; for 3 days the addition of a randomly generated number from 1 to 4 to the original PM_2.5_ concentration for each day; and for 2 days the subtraction of a random number between 6 and 30, that was identified as 13, to the original PM_2.5_ concentration for each day. The Excel function RANDBETWEEN was used to generate the random numbers that were used to either add or subtract a defined μg/m^3^ for each day. For the analysis the counterfactual, or policy scenario, assumes that PM_2.5_ concentrations are equal to an annual mean of 10 μg/m^3^, which represents the WHO annual PM_2.5_ Air Quality Guideline (AQG) value [13]. We further considered additional policy scenarios assuming alternative counterfactual concentrations of 5, 12, and 25 μg/m^3^.

#### 2.1.2. Population Data

For both, Subregion 1 and Subregion 2, we defined the population as being the total number of individuals older than 29 years of age; as we note below, this age strata corresponds to the age range considered in the epidemiologic study. Within the analysis, population data for Subregion 1 represents the total population of adults older than or equal to 30 years of age for the years 2004 – 2006 in Budapest, Hungary, which was defined as 1,156,588 [12]. This equated to ~68% of the population of Budapest, which is 1,690,109. Subregion 2 population data was randomly generated by first adding individuals to the total population of Subregion 1 (i.e., 1,690,109) and selecting a random number between 40% and 60% (51% was identified), resulting in a new total population of 2,556,266. Then, based on the total population, the percentage of the population older than or equal to 30 years of age was identified by generating a random number between 50% and 68% (54% was identified), this equated to a population of 1,391,237. By selecting a range of 50% to 68% when identifying the percent of adults within the population resulted in a younger population in Subregion 2 compared to Subregion 1. The Excel function RANDBETWEEN was used to generate the random numbers.

#### 2.1.3. Mortality Rate

The mortality rate, or baseline incidence rate, used for the analysis represents the total number of deaths for all-natural causes per year in each Subregion. Health data used in this analysis are based on mortality rates that were obtained for Budapest, Hungary for the years 2004 – 2006 [12]. For Subregion 1, the exact mortality rate for Budapest of 940 (per 100,000) was used. For Subregion 2, the mortality rate from Budapest was randomly modified to be lower to correspond to the younger population distribution of the region. To identify this rate, a random number between 700 (per 100,000) and 1,000 (per 100,000) was obtained, which was selected to be 830 (per 100,000). The Excel function RANDBETWEEN was used to generate the random number.

#### 2.1.4. Beta Coefficient

The beta coefficient and corresponding standard error were derived from the results of Hoek et al. [14], a meta-analysis of 11 cohort studies spanning the U.S., Canada, and Europe that examined the relationship between long-term PM_2.5_ exposure and all-cause mortality. The pooled estimate across the 11 cohorts for PM_2.5_ corresponded to a Relative Risk (RR) of 1.062 (95% Confidence Interval [CI]: 1.040–1.083) for a 10 μg/m^3^ increase in annual PM_2.5_ concentrations.

### 2.2. Steps in performing the analysis in AirQ+

In AirQ+, in order to calculate the premature deaths resulting from improved air quality levels, we need to perform an “Impact Evaluation”.

The steps to perform an “Impact Evaluation” first consist of importing the air pollution daily data. In our example, the Import Air Quality Data command of AirQ+ produces an arithmetic mean PM_2.5_ concentration for both regions combined of 27.50 μg/m^3^, with an arithmetic mean of 25.47 μg/m^3^ for Subregion 1 and 29.53 μg/m^3^ Subregion 2. After defining the exposed population (i.e., Subregion 1 = 1,156,588; Subregion 2 = 1,391,237) an overall population-weighted PM_2.5_ concentration of 27.69 μg/m^3^ is calculated and used for subsequent calculations (available in the Detailed Results tab). The population-weighted PM_2.5_ concentration is 25.47 μg/m^3^ for Subregion 1 and 29.53 μg/m^3^ for Subregion 2. The The average concentration is given as a population-weighted concentration and as the arithmetic mean of daily values over the three-year period 2004–2006. To run an ‘*Impact Evaluation’* the following steps are taken to enter the data:

1. Enter the mortality rate, which for this analysis represents deaths from all-natural causes for adults (≥ 30 years), per 100,000 population):
  - Mortality incidence in Subregion 1: 940
  - Mortality incidence in Subregion 2: 830
2. Enter the population of adults (≥ 30 years of age) in each location:
  - Subregion 1: 1,156,588;
  - Subregion 2: 1,391,237
3. Enter the risk estimate from an epidemiologic study for all-cause mortality for adults (≥ 30 years of age):
  - Relative Risk (RR) = 1.062 (95% CI 1.040–1.083) from Hoek et al. [14]
4. Define counterfactual value to reduce PM_2.5_ concentrations to:
  - WHO AQG value of 10 μg/m^3^
  - Sensitivity Analyses: 5, 12, and 25 μg/m^3^

**Figure 1.**
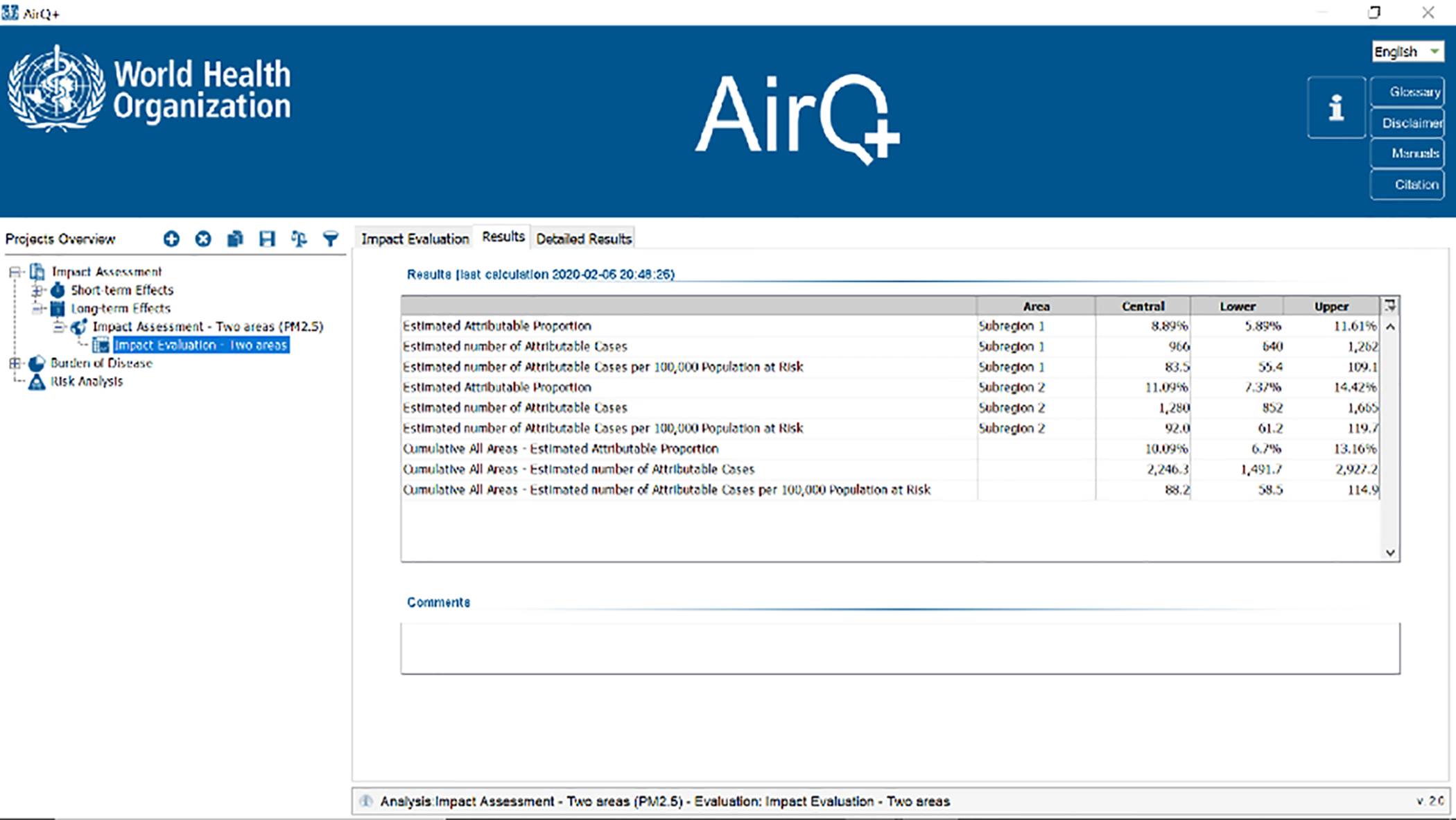
Screenshot of results generated by AirQ+.

Once the analysis is run, the results are populated in the Detailed Results tab (Figure 1).

### 2.3. Steps to performing the analysis in BenMAP-CE

**Figure 2.**
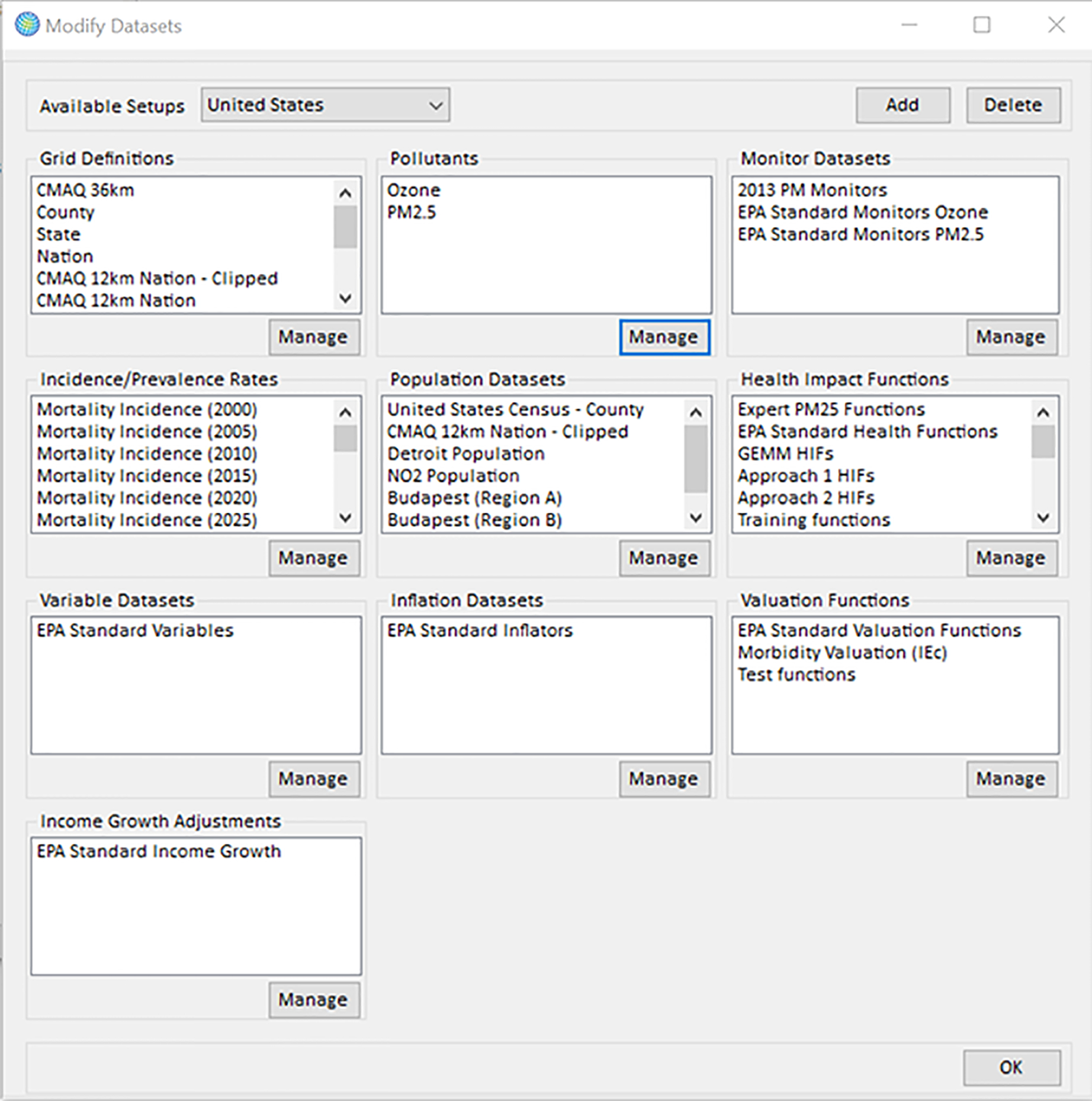
BenMAP – CE setup window.

The BenMAP-CE program is preconfigured to perform PM_2.5_ and Ozone health impact analyses in the U.S. Analyses for other regions, including Budapest, require users to update the configuration, as described in Sacks et al. [8]. In brief, users load data including: (1) the geographic location of the analysis and the air quality modeling or monitoring domain (i.e, the “grid”), saved as a shapefile (*.shp); (2) the population counts for the domain, stratified by age, sex, race and ethnicity; (3) the baseline rates of death and disease for each health endpoint quantified (in this case, mortality) stratified by age, sex, race and ethnicity; (4) the health impact function, which incorporates the concentration-response parameter, as described above. All of the information preloaded within BenMAP – CE as discussed in Table 1, can be observed in the BenMAP – CE setup window (Figure 2).

**Figure 3.**
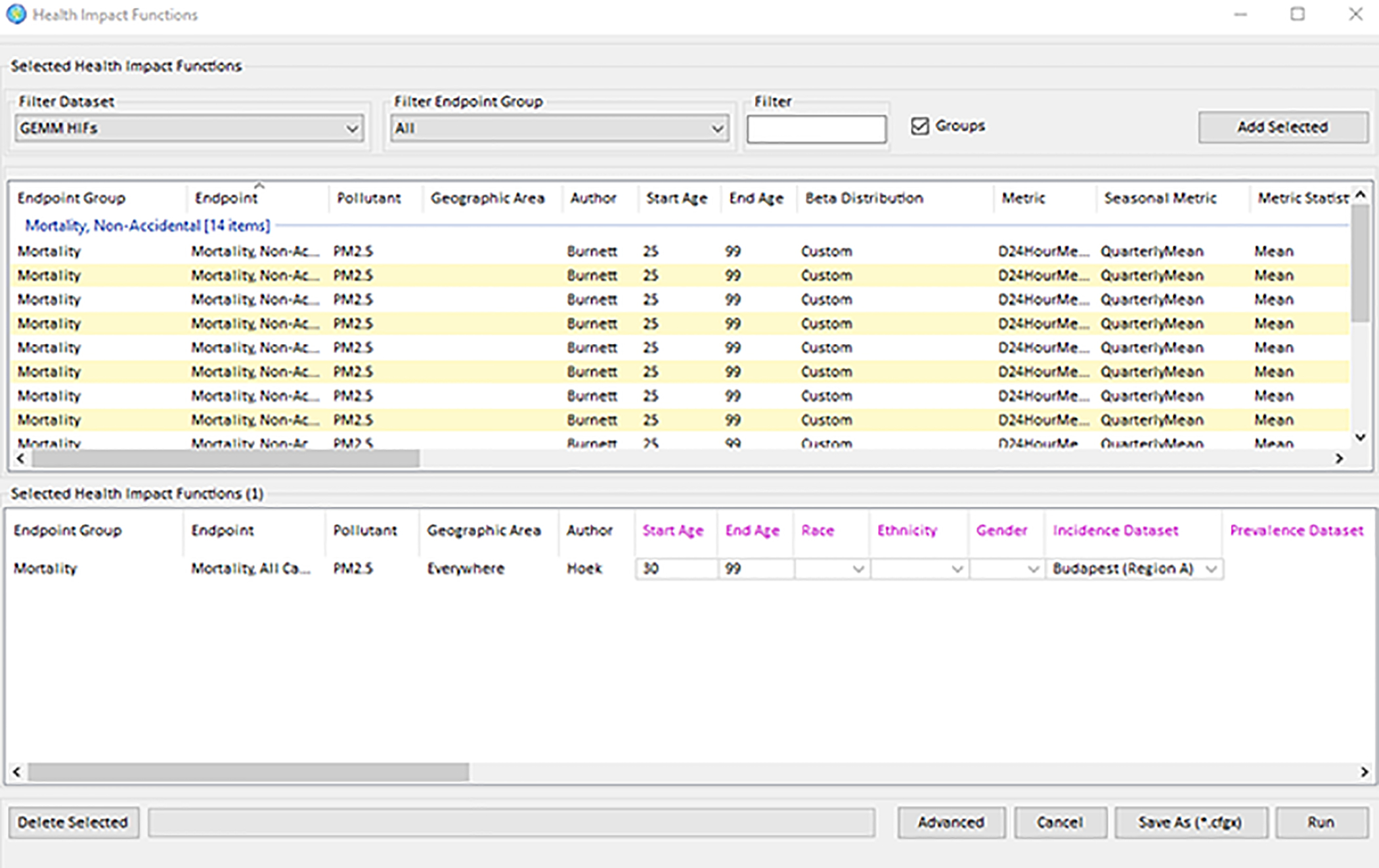
BenMAP – CE health impact function selection window.

Upon configuring the program, the user next performs the analysis as follows, using the input parameters as specified above:

1. Selecting the air quality “baseline” (i.e., pre-policy) and “control” (i.e., post-policy or post-rollback) air quality input files. The program next depicts the change in air quality using the built-in Geographic Information System.
2. Selecting the desired health impact functions and the corresponding population (Figure 3) The program then calculates and reports tabular and GIS results.

Because this analysis considers multiple scenarios of reducing PM_2.5_ concentrations to different counterfactual values (i.e., 5, 10, 12, and 25 μg/m^3^), we ran the BenMAP-CE program in batch mode using its scripting language as described in Sacks et al. [8].

## 3. Results

Across the two Subregions the average annual PM_2.5_ concentration was 27.50 μg/m^3^, which equates to a population-weighted concentration of 27.69 μg/m^3^. When examining each of the Subregions separately, Subregion 1 was found to have an arithmetic mean PM_2.5_ concentration of 25.47 μg/m^3^ while Subregion 2 was found to have an arithmetic mean PM_2.5_ concentration of 29.53 μg/m^3^.

When comparing the results of the analysis, both BenMAP – CE and AirQ+ calculated almost identical results for the central estimates at the integer level with minimal differences at the decimal digits level (Table 3 and Table 4). In addition to the main result, or central estimate, each tool also reports confidence intervals that bound the main result. AirQ+ calculates the 95% confidence intervals around the estimated health impacts using the 95% confidence interval associated with the risk estimate from the published epidemiologic study used in the analysis (e.g., 95% CI: 1.040–1.083 from Hoek et al. [14]). From the 95% confidence interval it is possible to calculate the corresponding standard error. Assuming a normal distribution, AirQ+ uses the standard error to estimate the uncertainty around the main result. In contrast, BenMAP-CE performs a Monte Carlo analysis, sampling the standard error reported in the epidemiologic study from which the beta coefficient is used. Thus, this confidence interval reflects statistical uncertainty in the epidemiologic study, but not other sources of uncertainty associated with the remaining input parameters. While U.S. EPA commonly reports counts of air pollution-attributable deaths and illnesses rounded to two significant figures, we report unrounded values to demonstrate the consistency of the results across the two tools. The slight difference in the calculation of confidence intervals between the two tools did not result in significant differences (less than 1%) in the reported 95% confidence intervals (Table 3 and Table 4).

In the main analysis where a counterfactual of 10 μg/m^3^ was used, which equated to the WHO AQG annual PM_2.5_ value, the results from Subregion 1 and Subregion 2 indicate that approximately 965, and 1,280 premature deaths are attributable to long-term PM_2.5_ exposure, respectively. Therefore, reducing PM_2.5_ concentrations in Subregion 1 and Subregion 2 to an annual PM_2.5_ concentration of 10 μg/m^3^ would reduce the total number of premature deaths attributed to long-term PM_2.5_ exposure.

In addition to the estimated number of attributable cases, which represents the main result from both tools, each allows for the calculation of additional results that give users greater insight into the estimated health impacts. For AirQ+, the tool directly calculates these additional values and includes the estimated attributable proportion of deaths from long-term PM_2.5_ exposure as well as the estimated number of attributable cases per 100,000 population at risk (Table 3). The estimated attributable proportion is a population-normalized value that reflects the percentage of total all-cause deaths that are attributable to PM_2.5_ exposure. The estimated number of attributable cases per 100,000 population at risk is also population-normalized and can be useful when comparing the health impacts attributable to air pollution across locations of different population sizes. While BenMAP – CE does not provide this information as an output in the results, it can easily be calculated using the input parameters for the analysis along with the main result presented from the analysis, i.e., the estimated number of attributable cases. As depicted in Table 3, in Subregion 1 the estimated attributable proportion is 8.9% and in Subregion 2, 11.1%, while the estimated number of attributable cases per 100,000 population at risk is approximately 83.5 in Subregion 1, and 92.0 in Subregion 2.

### 3.1. Sensitivity Analysis

When conducting sensitivity analyses using alternative counterfactual values representing annual PM_2.5_ concentrations of 5, 12, and 25 μg/m^3^, consistent with the main analysis, both BenMAP – CE and AirQ+ produced similar results. As the counterfactual value was reduced, the number of premature deaths attributed to long-term PM_2.5_ exposure increased, reflecting the increase in the difference between the baseline annual PM_2.5_ concentrations and the counterfactual PM_2.5_ concentration in both Subregion 1 and Subregion 2 (Table 2). Reducing annual PM_2.5_ concentrations to the counterfactual values of 25, 12, and 5 μg/m^3^ was estimated in Subregion 1 to equate to approximately 30, 850, and 1260 premature deaths, respectively, and in Subregion 2 310, 1150, and 1580 premature deaths, respectively.

## 4. Discussion

By using a common dataset, the main analysis, and sensitivity analyses, demonstrated that BenMAP – CE and AirQ+ produce similar results in the process of estimating the public health impact of poor air quality. The analyses further confirm that the underlying methodology used by each tool is consistent and that each tool can be used with confidence to estimate the public health impacts attributed to air pollution. Although the results obtained using both tools are similar, it is important to recognize the strengths and limitations of each tool as researchers or risk assessors embark on efforts to quantify air pollution-related health and economic impacts.

There are numerous strengths and benefits to using BenMAP – CE and AirQ+ individually, but each was originally developed for a different purpose, which is reflected in the different features of each tool. BenMAP – CE was developed for the purpose of supporting policy-related risk and benefits analyses in the process of promulgating environmental regulations at the U.S. EPA, while AirQ+ was developed as a decision support tool that also has a considerable educational focus for public health authorities [8]. Even with the original difference in the purpose of each tool, it is important to note the commonality amongst them and that both: (1) come with an extensive history of development and maintenance that have directly contributed to the large communities of users worldwide; (2) can estimate health impacts attributed to short-term (i.e., daily) and long-term (i.e., yearly) air pollution exposures; (3) can estimate impacts for a range of health endpoints and for different pollutants (see Table 1); and (4) offer features that are unique to each tool. Specifically, BenMAP – CE can estimate the potential economic impact associated with air pollution-related health impacts, which is a functionality not contained within AirQ+. However, AirQ+ offers the ability to estimate the health impacts attributed to household air pollution (i.e., due to solid fuel use) and an assessment of the cancer risk associated with some air pollutants, both of which are not possible in the current version of BenMAP – CE, but could potentially be included within the tool in the future. BenMAP – CE is designed to support U.S. federal, state and local air quality policies, allowing users to assess health and economic impacts over time and space using either pre-loaded or user-specified input parameters (Table 1). Similar to BenMAP – CE, AirQ+ can also be used to estimate health impacts at different spatial scales, and therefore be used to inform various potential air quality actions. Both BenMAP-CE and AirQ+ are useful tools for estimating the health impacts of poor air quality with the decision regarding which tool to use at the discretion of the user based on both data availability (e.g., type and resolution of air quality data) and the research question to be examined.

Although there are commonalities between BenMAP – CE and AirQ+, the differentiation in the original purpose of both tools has factored into the process used to determine the types of information preloaded within each. This is most prominently reflected in the health impact functions available in both tools that the user can select to estimate health impacts. Because BenMAP – CE is used extensively in the rule-making process for various environmental regulations, with a prominent role within the review process of the National Ambient Air Quality Standards (NAAQS), a more rigorous approach is taken in determining the health endpoints to estimate and, subsequently the selection of epidemiologic studies to be used to derive health impact functions. In selecting the health endpoints to quantify, the U.S. EPA relies heavily on the scientific evidence evaluated within the Integrated Science Assessments (ISAs), which form the scientific basis of the NAAQS. The ISAs represent a rigorous evaluation of the scientific evidence spanning epidemiologic, experimental (animal toxicological and controlled human exposure), dosimetry, exposure, atmospheric chemistry, and welfare effects studies using a weight-of-evidence approach to assess the causal nature of relationships between criteria pollutant exposures and health and welfare effects [15]. It is within the ISAs that the U.S. EPA conveys their overall conclusions on the degree to which the scientific evidence supports a causal relationship between an air pollutant exposure and health effect category (e.g., respiratory effects, mortality, etc.). The conclusions for each of the health effect categories evaluated directly informs the health endpoints considered for inclusion within BenMAP – CE. Specifically, for those health effect categories where the ISA concludes that a “causal relationship” or a “likely to be causal relationship” exists, the U.S. EPA further evaluates the available epidemiologic studies that formed the basis of these conclusions to identify the studies that could be used to derive health impact functions. Within this additional assessment of epidemiologic studies, the U.S. EPA is evaluating whether the additional pieces of information needed to derive a health impact function from an epidemiologic study are readily available, such as data that corresponds to the population examined within the study (e.g., total population ≥ 65 years of age) and the baseline incidence rate for the health effect evaluated (e.g., mortality rate for the population ≥ 65 years of age).

Compared to BenMAP – CE, AirQ+ was developed with a target audience of public health specialists in mind rather than policy analysts. As a result, although WHO relied heavily on the scientific evidence evaluated within expert groups in determining the air pollutant – health outcome relationships to quantify, it also includes features that are supportive of research activities in specific scientific areas. As a result, contrary to the approach used in BenMAP – CE for selecting the health outcomes to quantify, AirQ+ quantifies the impacts for some air pollutant – health outcome relationships where the evidence base is not as strong, specifically NO_2_, BC, and long-term ozone exposure. The philosophy behind WHO’s incorporation of these additional air pollutants and corresponding health impact functions into AirQ+ is that they can aid in identifying potential research gaps and open discussion not only with the scientific community, but also with users on the limits and benefits that should be taken into account when performing non-mainstream analyses.

While the overall process of estimating health impacts is similar between BenMAP – CE and AirQ+, there are differences in the resolution of information used in both tools, which is a function of BenMAP – CE containing GIS components. As a result, the analyses conducted within AirQ+ are at a much coarser spatial resolution, often over the spatial domain of an entire city or country. This differs from BenMAP – CE where it is possible to conduct analyses for an entire city or country, but at the grid cell level, as small as 1km × 1km, which can allow for a detailed analysis of how both exposures and health impacts vary across a geographic location.

In addition to the main features of BenMAP – CE and AirQ+, both contain additional features that are unique to each tool. For BenMAP – CE this includes the Global Burden of Disease (GBD) Rollback Tool and Popsim. The Global Burden of Disease (GBD) Rollback Tool has contributed to the ability of BenMAP – CE to expand its reach to international audiences. This easy to use tool that is embedded within BenMAP – CE allows for users to estimate the potential health and economic benefits (i.e., reductions in mortality) of improving air quality in any country or region worldwide using air quality, population, baseline health and concentration-response parameters from the GBD project. Within the tool, users can estimate the number of PM_2.5_-attributable premature deaths that could be reduced through “rolling back” concentrations by either: a fixed air quality increment (e.g., 5 μg/m3); a proportion (e.g., 5%); or down to various air quality standards (e.g. the U.S. EPA Particulate Matter NAAQS) or guidelines (e.g. the WHO AQG). Users can also estimate the economic value of these PM_2.5_-attributable premature deaths using a country-specific Value of Statistical Life. The GBD Rollback Tool is advantageous to users located in countries where it is difficult to obtain all of the underlying data needed to estimate health and economic impacts, such as baseline incidence rates and population data, the ability to gain a better understanding of the potential public health benefits of improving air quality.

The second feature within BenMAP – CE, Popsim, uses a life table to quantify the number of PM_2.5_-attributable life years gained, PM_2.5_-attributable deaths avoided, and improved life expectancy at birth as a result of reducing PM_2.5_ concentrations in the U.S. This tool uses country-specific life tables, thus accounting for the between-country variability in age-specific death rates. In contrast to the core BenMAP-CE program that is operated for a specific recent or future year, Popsim estimates year-to-year changes in the risk of death over a 50-to-100 year time horizon.

Compared to BenMAP – CE, AirQ+ has the added benefit of being able to conduct analyses to estimate the health impacts for different air pollution exposures that cannot be evaluated using BenMAP – CE. As is noted in the GBD project, household air pollution represents one of the top 10 risks to health worldwide, particularly in developing countries [1]. AirQ+ allows users to estimate the health impacts attributed to household air pollution through the inclusion of health impact functions for solid fuel use. The risk estimates used for household air pollution are based on solid fuel use, which is an area of scientific development that in the future may produce new methods that could potentially be included in future versions of AirQ+. Additionally, it is well characterized that air pollution contains many pollutants that have been classified as carcinogens [16]. Building off this scientific evidence, AirQ+ contains unit risk values for arsenic, benzene, benzo[a]pyrene, chromium (VI), nickel and vinyl chloride, allowing users to estimate cancer risks.

BenMAP – CE and AirQ+ have played leading roles in demonstrating the health and economic impacts of poor air quality, and directly contributed to the development of environmental actions to improve air quality. However, both tools continue to evolve, and institute features to expand upon their use and increase the sophistication of analyses than can be conducted. For example, AirQ+ is going through a new phase of development to increase its integration within WHO for all their ongoing air pollution activities, in particular for burden and impact analyses [17]. AirQ+ undergoes continuous enhancements, mainly related to modifications that reflect the scientific advice from experts and the feedback received from users. This includes additional documents to clarify definitions and input of data, new user-friendly components for the calculation of DALYs and economic impacts, and additional examples of calculations. Additionally, efforts have been undertaken to increase the use of AirQ+ worldwide through the recent release of German and Spanish versions and an additional module for the economic assessment of air pollution, taking into account suggestions from expert consultations [18]. BenMAP – CE continues to release updated versions of the tool with the most recent version released in March 2019, and ongoing efforts to institute new functionality into the tool, such as the estimation of health impacts attributed to multipollutant exposures. Advancements in BenMAP – CE have been facilitated by the tool being an open source platform and the constant engagement with the user community through direct user feedback and the BenMAP – CE user forum (https://forum.benmap.org). It is through the continued evolution of BenMAP – CE and AirQ+ and the institution of new and innovative features that both will continue to be at the forefront of research and policy efforts to estimate the public health impact of air pollution.

## Figures and Tables

**Table 1.** The different types of preloaded data and user provided data within BenMAP – CE and AirQ+.

	BenMAP – CE		AirQ+	
	Preloaded Data	User Provided Data	Preloaded Data	User Provided Data
Pollutants^1^	PM_2.5_ Ozone	User can conduct analyses for other pollutants if data provided as noted within this table	PM_2.5_ PM_10_ Ozone Nitrogen Dioxide (NO_2_) Black Carbon (BC) Solid Fuel Use	User can conduct analyses for other pollutants if data provided as noted within this table
Air Quality	Year 2000–2013 PM2.5 and ozone monitoring data for the contiguous U.S.	Import .csv or .xlsx file specifying air quality modeling or monitoring data	n.a.^2^	Import .csv file with air quality data for geographic area(s) of interest
Population	U.S. population projections from 2000 to 2050 in 1-year increments stratified by sex/age/race/ethnicity at 12 km grid cells	Import .csv or .xlsx file specifying sex/age/race/ethnicity for a defined population assigned by grid cell	n.a.	Import .csv file with population data for geographic area(s) of interest
Baseline Rate of Deaths and Illnesses	Cause-specific county-level death rates projected from 2000 – 2060 in 5-year increments Hospital and emergency department visit rates for 2013 at county- and state-level	Import .csv or .xlsx file specifying age/race/ethnicity stratified incidence rate assigned by grid cell for a defined geographic location	n.a.	Import .csv file with baseline rate of deaths for geographic area(s) of interest
β Coefficient	Over 100 PM_2.5_ and ozone health impact functions drawn from U.S. and Canadian studies. Endpoints include mortality, hospital admissions, emergency department visits, exacerbated asthma, acute respiratory symptoms, school/work loss days	Import .csv or .xlsx file specifying health impact function(s), including health endpoint, functional form, β coefficient, applicable age/sex/race/ethnicity information	Over 50 health impact functions spanning PM_2.5_, PM_10_, NO_2_, ozone, BC and solid fuel use drawn from European studies. Endpoints include all-cause and cause-specific mortality, postneonatal infant mortality, prevalence of bronchitis in children, incidence of chronic bronchitis in adults, incidence of asthma symptoms in asthmatic children, hospital admissions: CVD and respiratory diseases, Restricted activity days (RADs)	User can modify coefficients
Health Impact Function (HIF) Functional Form	Log-linear Logistic Global Burden of Disease (GBD) Integrated Exposure-Response (IER) Function	User can select various operators, variables, and population variables to define unique functions, including specifying different functions for different parts of an air quality distribution	Log-linear Linear-log Global Burden of Disease (GBD) Integrated Exposure-Response (IER) Function	n.a.
Distributions that can be Specified for Uncertainty Calculations	Normal Triangular Poisson Binomial Log Normal Uniform Exponential Geometric Weibull Gamma Logistic Beta Pareto Cauchy	Users can select a non-parametric custom distribution	n.a.	n.a.
Economic Values	Multiple cost-of-illness (COI) and willingness-to-pay (WTP) studies for each health endpoint quantified by health impact function	Import .csv or .xlsx file specifying COI or WTP function(s), including health endpoint and unit value	n.a.	n.a.
Additional Features	Global Burden of Disease (GBD) Rollback tool allows estimation of PM_2.5_ health impacts worldwide based on data from GBD study.	n.a.	Cancer Unit Risk Values for arsenic, benzene, benzo[a]pyrene, chromium (VI), nickel and vinyl chloride	User can modify coefficients

Adapted from Sacks et al. [8]

1 This row does not represent data contained within BenMAP – CE and AirQ+, but instead notes the pollutants for which some data is available (e.g., air quality data, health impact functions, etc.) within each tool that allows for an analysis to be conducted.

2 Although AirQ+ does not contain air quality data it does contain a conversion factors table to estimate PM_2.5_ concentrations from PM_10_ concentrations for over 100 countries. Additionally, AirQ+ contains information on the current WHO Air Quality Guidelines in order to conduct analyses that rollback air pollutant concentrations to various values to estimate health impacts the meeting current guidelines.

**Table 2.** Input parameters for each Subregion analysis conducted in BenMAP – CE and AirQ+.

Location	Main Analysis	Sensitivity Analysis
Subregion 1	Air Quality Data: Baseline: validated PM_2.5_ concentrations Control: PM_2.5_ concentrations rolled back to meet WHO AQG value of 10 μg/m3 Population Data: Adults ≥ 30 years of age Mortality Rate Data: 940 deaths (per 100,000) for all-natural causes Beta Coefficient: Hoek et al. [14]	Air Quality Data: Baseline: validated PM_2.5_ concentrations Control: PM_2.5_ concentrations rolled back to meet alternative values of 5, 12, and 25 μg/m3 Population Data: Adults ≥ 30 years of age Mortality Rate Data: 940 deaths (per 100,000) for all-natural causes Beta Coefficient: Hoek et al. [14]
Subregion 2	Air Quality Data: Baseline: PM_2.5_ concentrations randomly modified to be higher Control: PM_2.5_ concentrations rolled back to meet WHO AQG value of 10 μg/m3 Population Data: Adults ≥ 30 years of age Mortality Rate Data: 830 deaths (per 100,000) for all-natural causes Beta Coefficient: Hoek et al. [14]	Air Quality Data: Baseline: PM_2.5_ concentrations randomly modified to be higher Control: PM_2.5_ concentrations rolled back to meet alternative values of 5, 12, and 25 μg/m3 Population Data: Adults ≥ 30 years of age Mortality Rate Data: 830 deaths (per 100,000) for all-natural causes Beta Coefficient: Hoek et al. [14]

**Table 3.** Comparison of Estimated Benefits of Meeting the World Health Organization (WHO) Air Quality Guideline (AQG) Annual PM_2.5_ value of 10 μg/m^3^ Using BenMAP – CE and AirQ+.

	BenMAP – CE		Air Q+	
	Subregion 1	Subregion 2	Subregion 1	Subregion 2
**Estimated Attributable Proportion (%)**^1^	8.9 (6.0 – 11.7)	11.1 (7.5 – 14.5)	8.9 (5.9 – 11.6)	11.1 (7.4 – 14.4)
**Estimated Number of Attributable Cases**	965 (652 – 1,271)	1,278 (867 – 1,677)	966 (640 – 1,262)	1,280 (852 – 1,665)
**Estimated Number of Attributable Cases per 100,000 Population at Risk**^2^	83.5 (56.4 – 109.9)	91.9 (62.3 – 120.5)	83.6 (55.4 – 109.1)	92.0 (61.2 – 119.7)

Note: Results represent the central estimate and 95% confidence intervals.

1 Estimated Attributable Proportion (%) = (Estimated Number of Attributable Cases/[(Population per 100,000)*(Mortality Rate per 100,000)])

2 Estimated Number of Attributable Cases per 100,000 Population at Risk = (Estimated Number of Attributable Cases/Population)*100,000

**Table 4.** Comparison of Estimated Health Impacts Attributed to Meeting Alternative Annual PM_2.5_ Values of 5, 12, and 25 μg/m^3^ Using BenMAP – CE (a) and AirQ+ (b).

(a) BenMAP – CE Results						
	Cut-off 5 μg/m^3^		Cut-off 12 μg/m^3^		Cut-off 25 μg/m^3^	
	Subregion 1	Subregion 2	Subregion 1	Subregion 2	Subregion 1	Subregion 2
**Estimated Attributable Proportion (%)**	11.6 (7.86 – 15.17)	13.7 (9.3 – 17.9)	7.8 (5.2 – 10.3)	10.0 (6.8 – 13.1)	0.3 (0.2 – 0.4)	2.7 (1.8 – 3.6)
**Estimated Number of Attributable Cases**	1,258 (854 – 1,649)	1,582 (1,078 – 2,066)	846 (570 – 1,115)	1,154 (781 – 1,517)	31 (20 – 41)	310 (207 – 413)
**Estimated Number of Attributable Cases per 100,000 Population at Risk**	108.8 (73.8 – 142.6)	113.7 (77.5 – 148.5)	73.1 (49.3 – 96.4)	83.0 (56.2 – 109.1)	2.7 (1.8 – 3.5)	22.3 (14.9 – 29.7)

(b) AirQ+ Results						
	Cut-off 5 μg/m^3^		Cut-off 12 μg/m^3^		Cut-off 25 μg/m^3^	
	Subregion 1	Subregion 2	Subregion 1	Subregion 2	Subregion 1	Subregion 2
**Estimated Attributable Proportion (%)**	11.6 (7.7 – 15.1)	13.7 (9.2 – 17.8)	7.8 (5.2 – 10.2)	10.0 (6.6 – 13.1)	0.3 (0.2 – 0.4)	2.7 (1.8 – 3.6)
**Estimated Number of Attributable Cases**	1,260 (839 – 1638)	1,584 (1059 – 2051)	846 (560 – 1107)	1,156 (767 – 1506)	31 (20 – 41)	311 (203 – 410)
**Estimated Number of Attributable Cases per 100,000 Population at Risk**	108.9 (72.5 – 141.6)	113.9 (76.1 – 147.5)	73.2 (48.4 – 95.8)	83.1 (55.2 – 108.3)	2.7 (1.7 – 3.5)	22.3 (14.6 – 29.5)

Note: Results represent the central estimate and 95% confidence intervals.

1 Estimated Attributable Proportion (%) = (Estimated Number of Attributable Cases/[(Population per 100,000)*(Mortality Rate per 100,000)])

2 Estimated Number of Attributable Cases per 100,000 Population at Risk = (Estimated Number of Attributable Cases/Population)*100,000
